# Supplementary material for: Are carbon emissions trading and green financial instruments synergistic? -Comprehensive quantitative research based on content analysis
Source: PLoS One. 2024 Mar 7;19(3):e0298601. doi: 10.1371/journal.pone.0298601 (PMC10919601; doi:10.1371/journal.pone.0298601)
Supplement: S1 Data — (ZIP) [file pone.0298601.s001.zip › Data/地级市Z_F工作报告.zip!/╔╜╢1⁄2/╫═▓⌐╩╨/└┤╫╘╣1⁄2╓┌║┼í╛┬φ┐╦╩2╛▌═°í┐_2014.docx]

各位代表：

　　现在，我代表市人民政府，向大会作政府工作报告，请予审议，并请各位政协委员和其他列席会议的同志提出意见。

　　一、2013年工作回顾

　　刚刚过去的一年，面对错综复杂的国内外环境，全市上下在中共淄博市委的坚强领导下，深入贯彻落实党的十八大精神，牢牢把握主题主线和稳中求进的工作总基调，强化发展实干和以项目建设为纲的工作导向，扎实做好稳增长、调结构、促改革、惠民生各项工作，全市经济社会发展稳中有进，稳中向好。

　　——这是我们努力攻坚克难，经济发展质量和效益稳步提高的一年。预计全年地区生产总值实现3800亿元，比上年增长95%左右。实现公共财政预算收入27307亿元，增长711%。城市居民人均可支配收入和农民人均纯收入分别增长118%和13%。固定资产投资、社会消费品零售总额分别增长20%和131%。金融机构各项存贷款余额分别增长91%和101%。财政收入增幅同比有所回落，主要是宏观经济持续下行、落实结构性减税政策和主动减费降负等综合因素所致，2013年，全市地方税收突破200亿元，增长1102%，占公共财政预算收入的比重同比提高259个百分点，这说明全市经济增长的基础更加扎实，质量稳步提高。在保持合理发展速度的基础上，我们研究出台推进重大项目建设、扩大招商引资和扶持“双500强”企业发展的一系列政策措施，强化土地、资金等要素保障，全市384个亿元以上建设项目完成年度投资7761亿元，170个市重大项目完成投资350亿元。成功举办全国工商联直属商会经贸活动、赴港赴台经贸招商活动、浙商闽商走进淄博投资贸易洽谈活动、陶博会暨新材料技术论坛以及山东省产学研展洽会，成功签约一批重大项目，实际到位外来投资达283亿元，为下步发展积蓄了后劲和力量。

　　——这是我们加快转调创新，城市转型发展迈出坚实步伐的一年。出台促进企业技术改造的八条意见，预计全年完成技改投资850亿元，增长214%，占工业固定资产投资的比重达85%；大力实施创新驱动发展战略，六大战略性新兴产业利税、利润增幅分别高出规模以上工业65个和55个百分点，高新技术产业产值占比达到30%左右。建成MEMS研发中试平台、新材料展示和交易公共服务平台以及台北齐鲁科技园等海外孵化器，策划举办11期“创新大讲堂”，荣获“国家知识产权示范城市”称号。实施质量强市战略，鲁泰纺织荣获全国质量奖，东岳高分子荣获“省长质量奖”，35个企业荣获“市长质量奖”。加快产业布局调整，先进制造、新医药等重点产业功能区加速启动，淄博石嘴山工业园和部分境外资源开发项目建设进展顺利。深入实施现代服务业跨越发展工程，华润城市综合体、太保在线服务科技公司、淄博保税物流中心等一批重大项目加快推进，茂业天地、彩世界商业广场等大型商业设施建成开业，服务业比重提高17个百分点。粮食生产实现“十一连丰”，新型农业经营主体不断壮大，市级农业标准化生产基地发展到127万亩，山东黑牛特色产业园等被认定为全省高效生态特色产业园，现代农业发展水平不断提高。

　　——这是我们统筹城乡发展，新型城镇化扎实推进的一年。淄博新区建设全面提速，金融、商务等重要功能区块及水系、路网建设扎实推进，荣获全省首批绿色生态示范城区称号。文昌湖旅游度假区开发建设取得重大进展。南水北调续建配套主体工程、G205改造示范工程竣工，电网建设“十二五”投资目标提前两年完成。加大城乡环境综合整治力度，下大气力实施中心城区东部区域供暖热水管网改造等惠民工程，通过全国城市文明程度指数测评和国家卫生城市复审。14个省、市级示范镇完成固定资产投资402亿元，财政收入增长305%，产城融合发展迈出新步伐。开工建设农村住房164万户，完成危房改造1645户，改造农村公路3356公里，新解决1386万农村人口饮水安全问题。深入开展生态环境综合整治专项行动，加大环保执法力度，加强重点企业脱硫脱硝设施建设及城市污水管网改造，大气和水污染防治向纵深推进。扎实搞好国家森林城市和生态园林城市创建工作，完成造林面积137万亩、新增城市绿地316公顷，森林覆盖率和城市绿化覆盖率分别达到37%、443%。实施节能改造工程项目132项，节能减排目标全面完成。坚持保障科学发展与提高用地效率并重，荣获“全国国土资源节约集约模范市”称号。

　　——这是我们坚持以人为本，民生社会建设取得显著成效的一年。全面落实国家和省出台的各项民生政策，民生建设支出占财政总支出的比重达到57%。推进创业带动就业工作，新增城镇就业和农业转移人口就业分别完成年度计划的1977%和1464%，保障性住房建设完成省下达任务的1263%。城乡居民养老、医疗保险顺利整合，企业退休人员养老金、失业保险金、城乡居民基础养老金以及城乡低保、农村五保、城市“三无”对象保障、孤儿养育、城乡大病医疗救助等保障救助标准持续提高。探索建立临时救助制度，对城乡2896户特困家庭实施特别救济。启动“十万农户脱贫奔康工程”，组织实施扶贫开发和太河水库水源地保护区生态经济项目212项，贫困人口人均增收1157元，376万户、912万人实现脱贫。中小学办学条件标准化及校舍安全工程进展顺利，全面完成第一期学前教育三年行动计划，教育教学质量显著提高。不断深化医药卫生体制改革、文化体制改革，市级医疗机构对口支援欠发达区县闯出新路子，“十艺节”筹办和参演工作实现双丰收，十二届全运会参赛取得历史最好成绩，我市荣获“文化强省建设先进市”称号。人口自然增长率控制在3‰以内，低生育水平保持稳定。双拥共建工作取得新成效。扎实构建社会治理“三四二”格局，狠抓社会治安综合治理，毫不放松地加强安全生产和食品药品监管工作，着力加强应急管理能力建设，安全生产事故起数、死亡人数分别下降443%、154%，社会大局保持和谐稳定。统计、审计、物价、广播电视、民族宗教、外事、侨务、对台、防震减灾、气象、人防、史志、档案、关心下一代、妇女儿童、老龄、慈善、残疾人、红十字、援藏等各项工作都取得了新成绩，实现了新发展。

　　——这是我们积极改革创新，区域发展活力不断增强的一年。抓住列入全国老工业基地调整改造规划、全国资源型城市和省会城市群经济圈重大机遇，借势借力加快发展。齐鲁股权交易中心成功实施公司制改造，挂牌企业达到291家，直接和间接融资额超过100亿元。东营银行、青岛银行等外埠银行入驻淄博，高新区企业债券、市城市资产运营公司债券获批发行。出台《淄博市民间融资管理暂行办法》，市民间融资登记服务中心建成运营，整顿金融秩序优化金融生态环境专项行动取得明显成效。财政管理体制改革、“营改增”试点改革顺利推进。组建市公共资源交易平台。全市35%左右的农村家庭承包土地完成确权登记颁证工作。出台全面优化营商环境的一揽子意见，市级行政审批事项精简418%，建设项目“联审代办”和“一费制”启动实施，行政服务效能明显提高，有效激发了创业创新活力。

　　一年来，我们高度重视加强政府自身建设。认真执行市人大及其常委会的决议、决定，自觉接受人大、政协和社会各界监督，主动听取各民主党派、工商联和社会各界意见，认真办理人大代表建议和政协委员提案。认真贯彻中央和省市委关于改进工作作风、密切联系群众的各项规定，严格落实党风廉政建设责任制，倡俭治奢、倡勤治懒，改进服务、提高效能。积极推行“两条线”工作法，着力突破重点、打造亮点，办成了一批打基础、利长远的大事、实事。通过开办“市长市民面对面”节目等有效方式，倾听群众呼声，改进政府工作，解决实际问题，树立了为民、务实、清廉的良好形象。

　　各位代表，过去一年全市经济社会发展所取得的成绩来之不易。成绩的取得，是省委、省政府和市委正确领导的结果，是市人大、市政协与社会各界有力监督和大力支持的结果，是全市广大干部群众团结一心、攻坚克难、创新实干的结果。市人大、市政协以及各民主党派、工商联围绕中心、服务大局，加强对重点项目建设、新型城镇化以及大气质量管控、水资源保护等重大问题的视察监督、专题调研和政策建议，对推动政府加强和改进相关工作，发挥了重要作用。在此，我代表市人民政府，向全市广大干部群众，向各民主党派、工商联、各人民团体和社会各界人士，向中央、省驻淄单位、驻淄部队和武警官兵、政法干警，向海内外所有关心支持淄博改革发展的同志们、朋友们，表示衷心的感谢并致以诚挚的敬意！

　　在充分肯定成绩的同时，我们也清醒地看到，我市经济社会发展还面临一些突出困难和问题。主要是：经济下行压力依然存在，内生增长动力不足，稳中向好的基础还不牢固；经济结构性矛盾突出，原料型和初加工工业比重偏大，服务业发展相对滞后，资源环境约束日益凸显；保障和改善民生压力加大，结构性就业矛盾比较突出，加强和创新社会治理面临许多新课题；政府职能存在“缺位、越位、错位”问题，营商环境离市场主体的期盼还有较大差距，等等。对这些困难和问题，我们高度重视，将采取更加扎实有效的措施，认真加以解决。

　　二、2014年目标任务和工作重点

　　今年是全面贯彻落实党的十八届三中全会精神、全面深化改革的第一年，是深入实施“十二五”规划的攻坚之年。今年政府工作总的指导思想是：全面贯彻落实党的十八大和十八届二中、三中全会精神以及习近平总书记视察山东时的重要讲话精神，坚持稳中求进工作总基调，坚持把改革创新贯穿于经济社会发展各个领域各个环节，与时俱进地丰富完善发展实干和以项目建设为纲的工作导向，在持续增长中打好转方式调结构攻坚战，着力激发市场活力，提高经济发展质量和效益，着力保障和改善民生，创新社会治理，促进经济持续健康发展，社会和谐稳定。

　　各位代表！经过全市上下艰苦不懈地努力，淄博老工业城市转型发展取得显著成就，全市新的竞争优势正在形成。但同时我们也清醒地看到，当前国内外环境正在发生深刻变化，传统发展路径已经越来越难以适应，淄博经济已经到了只有加速转型升级才能持续健康发展的关键阶段。

　　我们必须以转方式的主动、调结构的主动、改革开放的主动，赢得经济发展上的主动和区域竞争中的主动。当前及今后一个时期，我们要下决心摆脱传统发展路径的束缚，用腾笼换鸟、凤凰涅槃的思路和办法，集中力量打好转方式调结构攻坚战，加大传统产业改造提升力度，加快转移淘汰过剩产能，大力发展战略性新兴产业和现代服务业，增创发展新优势。拿出凤凰涅槃、浴火重生的勇气和胆识，拿出抓铁有痕、踏石留印的措施和办法，敢于忍受产业转型需要经历的阵痛，敢于承受可能带来的风险，凝聚共识，坚韧不拔，一抓到底。

　　基于以上分析，根据市委关于今年经济社会发展的总体部署，今年全市经济社会发展的预期目标建议为：地区生产总值增长9%左右，公共财政预算收入增长11%左右，固定资产投资增长18%左右，社会消费品零售总额增长13%左右，实际利用外资和进出口总额增长10%左右，城市居民人均可支配收入和农民人均纯收入均增长10%左右，人口自然增长率控制在64‰以内，城镇登记失业率控制在4%以内，全面完成节能减排约束性目标。提出这样的指标体系建议，主要是兼顾就业形势、市场状况、资源环境承载等因素，更好地引导各级各方面把精力集中到提高质量和效益上，集中到促进经济结构不断优化、内生动力不断增强上，集中到抓好打基础利长远的大事上。

　　重点抓好以下九个方面的工作：

　　（一）集中力量做好“加减乘”促转调的三篇文章。推动结构升级、转型发展，要立足淄博实际、把握内在规律，强基固本做“加法”，节能环保做“减法”，创新驱动做“乘法”，深层着力、精准发力，推动产业内部结构从价值链低端向价值链高端转变，三次产业结构从“二三一”向“三二一”转变。

　　做好加法，加速传统产业高端化、集群化。围绕再造传统产业新优势，规划实施主导产业“铸链”提升工程，重点抓好化工、机械、建材、冶金、轻工、医药、纺织七大传统主导产业链，以大规模技术改造为主要抓手，逐条制定改造提升方案，逐个企业策划落实技改项目，通过发挥基础优势自我补链、加强链上企业横向联合协同强链、引进国内外顶尖企业借力拉链等多种途径，广泛采用先进装备和先进工艺，拉长产业链条，发展高端、形成集群。要较大幅度增加技改专项资金，对各区县和有关职能部门进行技改专门考核，倒排工期、强力推进，力争“十二五”期间全市技改投入突破3000亿元，到2017年对传统产业普遍改造一遍。通过大力实施主导产业“铸链”提升工程，显著提高最终产品、绿色产品、高端产品比重，降低原材料和中间产品、高消耗高排放产品比重，大幅度提高传统产业的附加值和竞争力。到“十二五”末，全市规模以上万元工业增加值能耗比“十一五”末降低17%以上，工业增加值率比现有水平提高2个百分点以上，40%的重点企业主要技术装备达到国际先进水平，传统产业加速脱胎换骨、浴火重生。

　　做好减法，为高端产业发展腾出空间。我市结构性矛盾特别突出、资源约束特别严重，必须下大决心压减低端、腾笼换鸟，以能耗和污染排放的“减法”，助推企业重生和效益提升的“加法”。一是积极有序转移一批。对那些由于资源环境约束加大、劳动力成本不断上升而丧失比较优势的产能，积极实施有组织、有秩序、有效益的转移，通过有序转移，发展贸易、重组债务、降低成本，努力做到过剩产能转出去、能耗物耗降下来、企业效益升上去。充分发挥政府引领作用，策划建设好一批境外、市外淄博工业园区，研究转移腾空土地工业转商住等政策，对“走出去”的企业给予大力度的支持，确保企业不论转到哪里，总部都留在淄博，财税增长都体现在淄博。二是强力关停淘汰一批。确保提前一年完成“十二五”淘汰落后产能任务，全面完成节能减排年度目标。在此基础上，进一步强化环境、安全等标准的硬约束，分阶段、分行业逐步提高能耗和排放强制性标准，引导相关企业主动转产或退出。对那些包袱越来越重、越做越亏、确实没有改造治理前途的企业，排出关停的时间表，制定关停方案和维稳措施，在扎实做好职工安置和社保接续等工作、确保稳定的前提下，积极果断实施关停。三是严把源头控住一批。严格能耗和排污总量控制，对新建项目实施能耗和排放减量替代，对新增煤炭消耗的项目严格限批，逐步做到煤炭消费只减不增，坚决把住环保节能门槛。推进省级以上工业园区循环化改造，争创全国循环经济示范城市。

　　做好乘法，着力培植重大增长极。战略性新兴产业和现代服务业是支撑和引领转型升级的新的重大增长极，对转型升级具有乘数和倍增效应，要集中要素、集成政策，全力支持，加快构筑我市新的竞争优势。一是做大做强战略性新兴产业。切实把握核心技术这个命脉，抓住新的巨大市场需求这个关键，立足我市比较优势，以培植先进陶瓷、稀土材料、环保催化剂、新能源汽车等首批20条高新技术产业创新链为主要抓手，“主链”与“子链”相结合，高起点定位、高标准规划，明确发展方向和发展目标，组建创新联盟，实施创新发展项目，培育优势产业集群，占领制高点、掌握主动权。力争20条创新链销售收入年均增长30％左右，到“十二五”末，使新材料产业、精细化工产业成为3000亿级产业集群，新能源及节能环保装备产业、汽车及机电装备产业、电子信息和新医药产业成为千亿级产业集群。要把规划建设新能源、新环保等重点产业功能区，作为发展新兴产业、优化生产力布局的战略平台，引进和实施一批重大项目，促进生产要素集成，大幅提高园区经济占全市工业经济总量的比重。二是推动现代服务业跨越发展。真正把发展现代服务业摆到更加重要的战略位置上，拿出不亚于抓工业、甚至超出抓工业的重视程度、投入强度和工作力度，真抓、大抓现代服务业。优先发展金融保险、现代物流、工业设计、信息服务等淄博最需要、也最有条件做大做强的生产性服务业，集中力量抓好齐鲁股权交易中心、化工物流基地和新材料物流中心、山东工业设计研究院和山东工业设计学院等重大项目建设。搞好首批国家信息消费城市试点，支持骨干企业发展电子商务，加快改造提升传统服务业。以齐文化开发为龙头，大力发展文化旅游业。加快推进齐古城、魔幻聊斋园等一批重大项目建设，形成重点景区景点的集成优势，以“体验齐文化、游览古村落”为主题积极发展乡村旅游，围绕“吃住行游购娱”六大要素配套发展旅游服务业，培育壮大骨干旅行社，努力把淄博打造成有重要影响力和知名度的旅游目的地城市。大力发展社会养老、社区服务、医疗健康和教育培训等生活性服务业，满足群众多样化的需求。进一步完善和落实支持服务业发展的政策措施，增加和用好服务业引导资金，提高服务业在科学发展考核中的权重，全面促进服务业发展提速、比重提高、水平提升。力争到“十二五”末，使服务业占比达到43%，到2020年达到50%以上，基本形成现代服务业占主导地位的经济结构。

　　（二）努力促进经济持续健康发展。坚持把稳增长牢牢抓在手上，实现经济发展质量和效益稳步提高又不会带来后遗症的速度，为调结构、促改革、惠民生奠定更实基础。

　　大力增强内需拉动。一以贯之地加强重大项目建设，充分发挥投资的关键作用。通过大规模招商引资、大规模上新创新、大规模发展民营经济，在提高项目质量、扩大优质投资上狠下功夫，同时把那些单纯扩大上游产能、加剧资源环境承载压力的项目剔除和替换出去，既保持健康增长的强劲动力，又不加剧新的结构性矛盾。按照上述思路，今年初步确定全市重大项目200个左右，总投资1700亿元左右。要建立强有力的项目建设推进机制、要素保障机制和督促考核机制，确保项目建设顺利推进，为更长时期、更高质量的发展打下坚实基础。要重视释放有效需求，充分发挥消费的基础作用。完善社会保障体系，坚持按劳分配和按要素分配相统一，工资性收入增长与劳动生产率增长相适应，切实增加城乡居民收入，改善居民消费预期。健全鼓励消费的各项政策，落实带薪休假制度，构建扩大消费需求长效机制。

　　着力强化要素支撑。认真贯彻稳健的货币政策，加强政银企合作的制度建设，搭建更多承接信贷资金的优质载体，努力扩大信贷规模，优化信贷结构，提高金融运行效率，增强服务实体经济的能力。力争将我市存贷比尽快提高到全省平均水平。要积极引导企业利用多层次资本市场体系，到主板、创业板、“新三板”及齐鲁股权交易中心融资和挂牌交易，多渠道实施股权融资，提高直接融资比重。建立健全信息共享的风险预警机制、政银协同的风险处置机制、汰劣保优的“防火墙”机制，有效防范和化解金融风险，坚决守住不发生系统性、区域性风险的底线。推进由民间资本发起设立中小型银行，做大做强地方金融企业。大力加强社会征信体系建设，优化区域金融生态环境。坚持盘活存量，优化增量，加大土地综合整治、增减挂钩、工矿废弃地复垦、批而未用土地清理工作力度，进一步提高用地保障能力和效率。

　　扎实提高财政经济质量。认真贯彻积极的财政政策，深入推进结构性减税，突出抓好“营改增”扩面试点，减轻企业和社会税收负担。坚持依法治税、科学治税、公平治税，确保税收增加真实反映经济增长的成果，不断提高税收占财政收入的比重。改革财政支持发展的方式，提高财源建设水平。建设性财政资金更多地采取“拨改贴”、“拨改投”、“拨改保”等方式，通过贷款贴息、投资参股、担保增信，充分放大导向和杠杆效应，带动更多社会资金。建立“花钱必问效、无效必问责”的机制，市级对财政拨款达到规定额度的投资发展类项目，全部实行绩效目标管理。切实加强政府债务管理和风险防控工作。深化财税体制改革，加快构建全面规范、公开透明的预算制度，2014年试行公开市本级部门和“三公”经费预算，2015年公开市本级部门决算和“三公”经费决算。

　　（三）大力推进创新驱动发展。深入实施创新驱动发展战略，优化创新资源配置，提高区域创新效率，提升核心竞争力。

　　构建富有活力的区域创新体系。紧紧围绕科研投入成果化、科技成果产业化，更好地发挥市高新区的辐射带动作用，加快规划建设开放式创新中试基地，全面加强生物制药、精细化工和高分子材料、无机非金属材料等全市公共创新平台建设，充分发挥国家级孵化器和海外孵化器功能，大规模引进和转化国内外成熟的科研成果，提高区域协同创新和产学研协同创新水平。大力实施企业创新能力提升工程，鼓励企业增加研发投入，加强与一流研发机构的紧密型合作，增强关键核心技术获取能力，积极承接和实施重要科技专项，充分发挥创新主体作用。

　　构建富有效率的创新模式和机制。加快商业模式创新，充分用好齐鲁股权交易中心这一特有平台，聚合风险投资、创业投资，完善科技投资担保体系，构建创新市场与资本市场有机对接的机制，支持创新成长型中小企业大批量挂牌入市，促进科技成果快速资本化、产业化。按照市场化取向，改革政府对创新的支持方式，建立主要由市场决定项目设立、经费分配和成果评价的机制，进一步改进和加强对“创新发展500强”的扶持，促进创新资源高效配置。高标准推进知识产权示范市建设，深入实施标准化战略和质量强市战略，加大知识产权保护和创新发明奖励力度，把尊重劳动、尊重知识、尊重人才、尊重创造方针落到实处，切实形成全社会支持创新、参与创新的浓厚氛围。

　　强化人才智力支撑。探索实行国际通用的人才机制和政策，建设好科技创新人才、企业家和高技能人才三支队伍。建设高层次人才社区，解决好高端人才关注的创业、居住和子女教育等问题。鼓励企业采取技术入股、期权激励等措施，与海内外高层次人才和创新团队建立紧密合作关系。大规模引进国外智力，走出一条高效便捷的创新之路。大力发展职业教育，优化专业设置，深化校企合作，加强与国外知名职业院校合作办学，培养大批市场急需的高技能人才。弘扬鼓励创新、宽容失败的创新文化，为各类人才施展才能创造良好环境。

　　（四）扎实推进新型城镇化。认真贯彻落实中央城镇化工作会议精神，遵循规律、增速提质，加快推进以人为核心的城镇化。

　　坚持正确的城镇化方向。坚持从淄博实际出发，把建设提升城市组群作为城镇化的主体形态，集中力量发展壮大主城区，着力建设提升次城区和县城，切实优化“中心凸显、十字展开、组团特色、融合发展”的城镇化布局。以淄博新区建设为龙头，全面加快文化、商务、金融、水系、教育、医疗等重要功能区块的规划建设，大力培育高端服务业，确保三年成气候、五年大变样，充分彰显“新淄博·大都市”的气韵风貌。高水平构建城区间路网、绿色长廊和生态水系，切实增强主城区与次城区和县城在规划、建设、管理上的关联性和协调性，增强聚合产业、吸纳人口、综合服务功能，形成组团发展的新优势。加快文昌湖旅游度假区开发建设步伐，打造城乡统筹、生态文明示范区。高标准建设省市示范镇，有重点地建设产业集聚、用地集约、人口集中、延续历史文脉、地域特色鲜明的小城市。深入推进生态文明乡村建设工程，提高农村新型社区建设水平。

　　大力推进以人为核心的城镇化。把实现被征地农民和进城务工人员市民化作为新型城镇化的首要任务，按照中央要求，全面放开县域城镇落户限制，建立财政转移支付同农业转移人口市民化挂钩机制，加快实现社保、教育、医疗、住房保障等基本公共服务覆盖全部城镇常驻人口，加快实现同工同酬。健全城乡均等的公共就业服务机制和覆盖城乡的创业培训体系，提高农业转移人口在城镇稳定就业的能力。在一定时期内，继续保留进城落户农民原集体经济组织成员的权利和待遇不变，使其享受新型城镇化带来的双重好处。

　　提升城镇综合承载和服务功能。加快推进城市轨道交通、S102济青线淄博段改建和济青高速铁路淄博段、小清河淄博以下通航等重大项目的前期工作，组织实施一批骨干路网改造工程，打造现代交通体系。加快中石化济青二线淄博段建设，优化能源结构，稳定气源供应。继续推进坚强智能电网建设，实现全市智能配网全覆盖。抓好引太入张调蓄工程、第二环保电厂、城区供暖热水管网等一批重点项目建设，完善城市功能。高度重视搞好城市地下空间规划利用工作。搞好全国“智慧城市”试点，加快云计算中心建设，提高城市运行和管理的智能化水平。理顺城市管理体制，成立市城市管理委员会，实现网格化、精细化、长效化管理全覆盖。

　　积极融入省会城市群经济圈战略。认真对接“一圈一带”建设发展规划，高水平策划一批交通、能源、水利、生态重大项目，在推进济淄一体化发展中显著提升淄博的城市承载功能。充分利用省会城市教育、人才、科技、资本等要素资源富集的优势，畅通合作机制，推动我市转调创新。充分发挥齐鲁股权交易中心、淄博保税物流中心等重要平台的辐射功能，在服务全省发展大局中提升淄博的城市地位，增强淄博的凝聚力、辐射力、影响力。

　　（五）加快发展现代农业。按照“三个导向”的要求，加快转变农业发展方式，大力发展现代农业，促进农业增效、农民增收、农村生态提升。

　　以满足吃得好吃得安全为导向，大力发展优质安全农产品。实施粮食增产科技集成推广工程，确保2017年前建成全省首批吨粮市。实施农业标准化生产基地建设提升工程，加强农产品质量标准、安全检测和认证三大体系建设，从生产源头保障农产品质量安全。扩大“三品一标”农产品规模，大力发展精深加工，培育农业名优品牌。发挥供销社连接城乡的桥梁纽带作用，创新农产品流通方式和流通业态，提高现代农业的附加值和竞争力。

　　以缓解地少水缺的资源环境约束为导向，大力发展生态友好型农业。严守耕地红线，提高节约集约用地水平，推动循环农业、节水农业、生态农业向纵深发展。组织实施小清河淄博段以及淄河、孝妇河等中小河流治理工程，新建高标准节水灌溉农田18万亩，再解决10万农村人口的饮水安全问题。进一步抓好秸秆禁烧和深度转化利用工程，组织实施好中央和省投资农村沼气项目。加强农业面源污染治理，净化农产品产地环境。加强基层农技推广、重大动植物疫病防控和农业信息化建设，提高现代农业支撑条件。

　　以解决好地怎么种为导向，加快构建新型农业经营体系。深入推进农村产权制度改革，扎实做好农村集体土地确权颁证工作，搞好农村土地承包经营权、农村集体经营性建设用地等产权流转交易市场建设。鼓励农民以承包经营权入股发展产业化经营，鼓励和引导工商资本进入农业和农村。加快构建职业农民队伍，大力扶持家庭农场、专业大户、农民合作社、产业化龙头等新型农业经营主体，提高集约经营、规模经营、社会化服务水平。

　　（六）提高开放型经济发展水平。实行更加积极主动的开放战略，坚持对内对外开放相互促进、引资引智相互融合、引进来和走出去更好结合，以开放促改革、促发展、促转型。

　　大力加强招商引资工作。坚持把招商引资作为推动转型发展的重要支撑，围绕做好“加、减、乘”三篇文章的关键需求，策划和推进重大招商项目，着力实现主导产业“铸链”招商、战略性新兴产业招商和现代服务业招商新突破。坚持在外招商专业团队与市内服务团队两线配合、上下联动，积极拓展重点国家和地区的优质招商资源，大力加强与跨国公司以及全国工商联、浙商、闽商、台商、港商的联系合作，增强招商的针对性和成功率。认真落实国家放宽投资准入的政策，开辟绿色通道，保障要素供给，确保招商项目顺利落地建设，力争全年实际到位外来投资增长45%以上。

　　努力扩大对外贸易。继续深入实施以质取胜和市场多元化战略，全面落实促进贸易便利化、扩大出口的各项政策措施，做大做强装备制造、精细化工、纺织服装和有机农业等特色出口基地，大力发展服务贸易，扩大出口总量，优化出口结构；同时，注重发挥进口对结构调整的支撑作用，扩大重要资源能源、先进技术和关键设备进口。支持淄博保税物流中心加快申报建设综合保税区，创新加工贸易模式，打造有利于推动外向型产业集群发展的重大平台。按照国际化、市场化、专业化的思路，进一步办好陶博会和新材料技术论坛。

　　坚定不移实施“走出去”战略。支持有条件的企业到境外布局产业链，进行境外资源开发、优势产能转移、国际研发合作，鼓励企业和个人自担风险跨国投资、自由承揽工程和劳务合作项目，不断拓展发展空间。进一步为企业和个人“走出去”提供信息服务和政策引导，帮助其控制风险、规避损失。大力发展总部经济，加快培育本土跨国公司。

　　（七）大力加强生态淄博建设。生态建设关系淄博的生存和未来，既是重大环境问题、发展问题，也是重大民生问题、政治问题，必须痛下决心，综合施策，标本兼治，“三城同创”，努力打造全国生态文明先行示范区。

　　用铁的手腕强力狠抓环境保护工作。突出抓好大气污染防治各项重点工作。坚决贯彻国务院大气污染防治十条措施和省市行动计划，集中打好重点燃煤行业综合整治、扬尘污染治理、黄标车淘汰报废、露天矿山整治四场攻坚战，明确责任主体，落实治理措施，限期完成治理任务。大力调整能源结构，在建成区划定高污染燃料禁燃区，加快推进高耗能企业清洁能源置换，强力推进热电联产和集中供热。加大产业结构调整力度，严格环保准入门槛，严格执行大气污染物排放新标准，加强中心城区周边工业园区等重点区域污染治理，确保“蓝天白云、繁星闪烁”的天数明显增加。按照“治用保”的思路大力强化水环境治理。认真落实国务院《城镇排水与污水处理条例》，加大污染点源治理力度，抓好城镇污水处理厂及配套管网和人工湿地建设改造，严禁污水直排河道，确保主要河流水质稳定达标。健全全市环境安全防控和应急管理体系，加快市危险废物综合处置中心建设，深化重金属污染综合防治，毫不放松地抓好饮用水源地保护，确保环境安全。

　　下大气力优化城乡生态环境。加强绿色生态屏障建设，组织实施好森林围城、荒山绿化、路域水系绿化、农田林网等大环境绿化，深入开展城市绿荫行动，新增园林绿地面积300公顷以上，让所有城市裸露土地全部绿起来。坚持水网、路网、林网同步规划，生态、产业、交通同步提升。实施全域生态水系建设工程，全面推进孝妇河、淄河等重点流域综合整治。扎实推进“清水润城”工程，高水平规划建设黄土崖等一批湿地公园，提升城市滨水景观。落实最严格的水资源保护制度，推进洪雨水和中水资源化利用，全面关闭自备井，争创全国水生态文明城市。组织“矿山复绿”专项行动，着力抓好生态脆弱区的恢复和治理。深入开展农村环境“连片整治”，改善城乡环境面貌，加快城市森林化、乡村园林化。

　　构建齐抓共管防治污染的体制机制。建立领导干部生态环境保护责任制，层层签订污染防治责任书、“军令状”，建立党政同责、横到边、纵到底的责任体系。建立空气质量每月公开通报和排名制度，对未完成年度治理任务且环境质量列全市后三位的区县实施区域限批，对相关责任人员严厉问责。推行居民用电、用水、用气阶梯价格制度，健全促进节能减排的差别价格机制。要强化打击环境违法行为联合执法机制，对偷排偷放、违规建设、严重超标排放等违法行为，严惩重罚，决不放过！

　　（八）加强民生和社会建设。继续按照守住底线、突出重点、完善制度、引导舆论的思路，统筹做好改善民生各项工作，提高社会治理水平，让发展成果更多更公平地惠及全市人民。

　　做好就业和社会保障工作。大力鼓励创业创新，设立创业带动就业扶持资金，完善创业就业服务体系，突出做好以高校毕业生为重点的青年就业，统筹做好农村转移劳动力、城镇困难人员和退役军人就业，力争全年新增城镇就业和农村劳动力转移就业14万人，高校毕业生总体就业率达到85%以上。完善覆盖城乡的社会保障体系，确保整合后的城乡居民养老、医疗保险制度健康运行，稳步提高社会保障水平。统筹完善城乡低保救助制度，深入开展党员干部联系困难家庭工作，建立临时社会救助制度，解决好极贫特困人员的生产生活问题。加强保障性住房建设，年内建成保障性住房10000套。今年是“十万农户脱贫奔康工程”的攻坚年，要加大组织推进和政策扶持力度，推动行业扶贫、专项扶贫和社会扶贫合力攻坚，为实现三年脱贫奔康目标打下坚实基础。

　　提高社会事业发展水平。努力办好人民满意的教育，启动第二期学前教育三年行动计划，推进中小学办学条件标准化建设，加强中小学校长教师交流轮岗，推动普通高中优质特色发展，扩大优质教育资源覆盖面，进一步促进教育公平。继续深化医药卫生体制改革，深入推进县级公立医院和基层卫生机构综合改革，巩固基本药物制度，加快推进市中心医院西院区等重点项目建设，提高优质医疗资源供给能力。坚持计划生育基本国策，稳定低生育水平。加快文化体制改革，做大做强文化产业，增强城市文化软实力。搞好市体育中心等文体资源管理运营，积极备战23届省运会，促进群众体育和竞技体育全面发展。加强国防动员和双拥共建工作，推动军民融合式发展。做好关心下一代工作，健全农村“三留守”人员关爱服务体系，保障妇女、儿童、老年人合法权益。促进残疾人、慈善、红十字等各项事业健康发展。引入竞争机制，加大政府向社会组织购买服务力度，增强社会事业发展活力。

　　加强和创新社会治理。坚持系统治理、依法治理、综合治理，健全基层综合服务管理平台，深入实施网格化管理、信息化建设、社区化服务“三大工程”，进一步推动管理服务重心下移。健全安全生产责任体系、隐患排查治理和安全预防控制体系，全面提高本质安全水平，确保安全生产形势持续稳定。健全市、区县、镇办食品药品监管机构，强化全过程监管和责任追究，确保食品药品安全。着力提高社会治安立体化防控水平，严密防范、严厉打击各类违法犯罪活动，建设平安淄博。深化“平安行·你我他”行动，打好道路交通安全综合治理持久战。扎实做好第十一届村民委员会换届选举工作。

　　（九）全面深化经济体制改革。按照中央和省关于全面深化改革的统一部署，扎实有序推进各项改革，着力在激发经济内生动力、释放社会发展活力上下功夫、见实效。

　　加快转变政府职能。该放的权坚决放开放到位，大力推进工商登记便利化、统一市场准入制度及确立企业投资主体地位等各项改革，最大限度地减少对市场主体生产经营活动、一般投资项目和资质资格等的许可、审批，充分发挥市场配置资源的决定性作用，充分激发市场活力和创造力。该管的事必须管住管好，切实强化环境保护、安全生产、食品药品安全监管、社会治安、市场监管等职能，重拳打击各类违法违规行为，为城乡群众创造生态和谐宜居的生活环境，为各类市场主体营造公平竞争的市场环境。进一步创新行政管理方式，把拟县化管理、扩权强镇等各项改革措施落到实处，简政放权、理顺体制、提高效率。

　　大力发展民营经济。坚持“两个毫不动摇”，坚持权利平等、机会平等、规则平等，清理和废除对民营经济的各种不合理规定，清除各种隐性壁垒，落实好支持民间投资77条实施细则。鼓励民营企业参与国企改革，鼓励发展非公有资本控股的混合所有制企业。建设好市民间融资登记服务中心，为民间融资提供规范化、阳光化、法制化渠道。加强市中小企业服务中心及公共技术服务平台建设，组织实施好“专精特新”中小企业培育工程。健全民营经济权益保护、投诉处置机制，保证民营经济依法平等使用生产要素、公开公平公正参与市场竞争、同等受到法律保护。

　　扎实有序推进各项改革。深入学习贯彻党的十八届三中全会《决定》，坚持以解决制约科学发展的重大问题为导向，正确、准确、有序、协调推进各项改革。全面深化国有企业改革，以管资本为主加强国有资产监管，完善公司法人治理结构，推行职业经理人制度。有序推进事业单位分类改革，年内基本完成从事生产经营活动事业单位转为企业或社会组织。深化社会领域改革，积极落实被征地农民参加社会保障办法和城乡基本保险制度衔接办法。对那些中央有明确要求、我们有良好基础的改革事项，加快推进，率先突破，推动经济社会更有效率、更加公平、更可持续发展。

　　三、建设法治政府和服务型政府

　　全面深化改革，推进国家治理体系和治理能力现代化，对政府自身建设提出了极高的要求。我们必须加快转变政府职能，创新行政管理方式，加快法治政府和服务型政府建设步伐。

　　（一）大力提高依法行政水平。建立法治政府建设推进机制，善于用法治思维和法治方式深化改革、推动发展、化解矛盾，严格依照法定权限和法定程序行使权力、履行职责。制定《重大行政决策程序规定》，完善规范性文件、重大决策合法性审查机制，提高决策的科学化、民主化、法制化水平。深入开展“行政执法规范化建设年”活动，全面落实行政执法责任制和过错追究制。加强反腐倡廉制度建设，做到用制度管权、管人、管事。自觉接受人大、政协和社会各界监督，加强审计监督和行政监察，全面推进政务公开，确保权力在阳光下运行。

　　（二）大力提高政府执行力。坚持把狠抓落实作为政府工作的生命线，坚决贯彻中央和省、市委的各项决策部署，确保政令畅通，令行禁止。各级政府要带头过紧日子，严格执行《党政机关厉行节约反对浪费条例》等各项规定，大力压减一般性支出，把“约法三章”落到实处。要牢固树立“功成不必在我”和“政贵有恒”的发展观、政绩观，改革完善考核评价体系，多做打基础、利长远的工作。建立健全目标责任清晰、贯彻执行坚决、监督考核有力的工作落实推进机制，用强有力的约束力倒逼和推动执行力，真正以踏石留印、抓铁有痕的制度和决心，确保改革发展稳定任务落实。

　　（三）大力营造良好发展环境。按照中央和省市委部署，扎实深入开展党的群众路线教育实践活动，毫不松懈地反对“四风”，推进改进作风制度化、常态化。牢记全心全意为人民服务的宗旨，深入基层接地气、深入实际摸实情、深入群众听民意，扎扎实实多办、办好惠民利民的实事、好事。全面落实优化营商环境的一揽子意见，深入推进行政审批“两集中、两到位”改革以及建设项目“联审代办”和“一费制”改革，严肃查处行政不作为、乱作为、慢作为，维护市场主体合法权益。认真开展“纳税500强”企业评议经济管理部门和基层群众评议民生服务部门工作，倒逼各级各部门更好地改进作风、提高效能，以实实在在的服务和业绩取信于民。

　　各位代表！时代赋予重任，奋斗铸就辉煌。让我们紧密地团结在以习近平同志为总书记的党中央周围，在中共淄博市委的坚强领导下，全面贯彻落实党的十八届三中全会精神，解放思想、改革创新，锐意进取、埋头实干，为加快建成更高水平、更高质量的全面小康社会，创造淄博人民更加幸福美好的未来而努力奋斗！
